# Development and optimization of large-scale approaches to identify iron-related genes in *Aspergillus fumigatus*

**DOI:** 10.3389/fmicb.2025.1646661

**Published:** 2025-07-31

**Authors:** Clara Baldin, Ulrike Binder, Jakob Scheler, Ernst R. Werner, Fabio Gsaller, Michael J. Bromley, Hubertus Haas

**Affiliations:** ^1^Institute of Molecular Biology, Medical University of Innsbruck, Innsbruck, Austria; ^2^Department of Microbiology, University of Innsbruck, Innsbruck, Austria; ^3^Institute of Hygiene and Medical Microbiology, Medical University Innsbruck, Innsbruck, Austria; ^4^Institute of Biological Chemistry, Medical University of Innsbruck, Innsbruck, Austria; ^5^Manchester Fungal Infection Group, University of Manchester, Manchester, United Kingdom

**Keywords:** iron-related screenings, HTS, fungal pathogens, porphyrin fluorescence, high iron toxicity

## Abstract

Recent advancements in genetic engineering have enabled the creation of extensive mutant libraries across various species, driving the need for efficient screening methods to identify mutants of interest. In this study, we developed and optimized two rapid and straightforward screening techniques to identify genes involved in iron metabolism. Iron is an essential element for almost all organisms, and in pathogens, the ability to acquire iron from the environment and mitigate the toxic effects of intracellular iron often plays a crucial role in virulence. The first screening method exploits the autofluorescence property of porphyrins, while the second one is an optimization of growth assay on solid-agar suitable for large scale analyses. To validate these methods, we applied them to a recently published protein kinase deletion mutant library in *Aspergillus fumigatus*, a fungal pathogen that causes severe diseases in immunocompromised individuals. Our iron-specific screening approaches successfully identified strains with altered iron metabolism, including both previously known and novel mutants, generating a small set of genes that can serve as new targets for antifungal therapies. These methodologies provide the first large-scale tool for exploring iron metabolism-related genes and can be adapted for other organisms with available mutant libraries.

## Introduction

The most recent assessment of the “Global incidence and mortality of severe fungal disease” (Denning, 2024) reveals a critical rise in annual deaths caused by fungal-related illness, with an estimated 6.55 million people worldwide likely to develop life-threatening fungal infections each year. Among the most significant opportunistic fungal pathogens is *Aspergillus fumigatus*, responsible for the development of invasive aspergillosis and other severe manifestations, particularly in immunocompromised individuals (Bongomin et al., 2017; Latge and Chamilos, 2019; Denning, 2024). The growing incidence of invasive aspergillosis, along with increasing antifungal resistance, has been a catalyst for the WHO to call for the identification of new antifungals targeting novel pathways (Parums, 2022; WHO, 2025). In pathogenic fungi like *A. fumigatus*, iron is a crucial element not only as a cofactor for various cellular processes (Ramos-Alonso et al., 2020), as in most living organisms, but also due to its key role in pathogenicity (Bairwa et al., 2017; Pijuan et al., 2023). The ability to acquire iron from the environment and manage its intracellular levels is a critical determinant of the pathogen’s virulence (Misslinger et al., 2021).

To fully understand the complex genetic networks in fungal metabolism, large-scale genetic screening has emerged as a powerful tool (Furukawa et al., 2020; Ross and Santiago-Tirado, 2024; van Rhijn et al., 2024). Techniques like gene knockout library and RNA interference (RNAi) screening enable the systematic identification of genes involved in complex biological pathways (Mohr et al., 2010; Zugasti et al., 2016; Ross and Santiago-Tirado, 2024). These methods have successfully identified genes essential for survival under nutrient-limiting conditions or when facing host immune defenses in other fungal pathogens. Several other screenings consider the sensitivity of mutant strains to specific antimicrobial substances, and their synergistic or antagonistic effects.

In the context of iron metabolism, genome-wide screenings could uncover not only key players in iron acquisition and storage but also the regulatory networks that maintain iron homeostasis (Lesuisse et al., 2005). Coupled with transcriptomic and proteomic analyses, these screenings can provide a comprehensive view of how fungal pathogens respond to iron limitation and adapt to the iron-restricted environment of the host during infection (Caza and Kronstad, 2013; Bairwa et al., 2017). However, large scale approaches to identify iron-related genes have not been developed yet, due to the difficulties to upscale the already established assays.

*A. fumigatus* represents one of the species in which extensive studies have already been conducted to better understand the mechanisms underlying iron sensing, acquisition, and metabolism (Haas, 2012; Misslinger et al., 2018; Yap et al., 2023). To date, the primary control mechanism for iron sensing in *A. fumigatus* revolves around a regulatory loop involving two main transcription factors: SreA and HapX (Haas et al., 1999; Tanaka et al., 2002; Hortschansky et al., 2007; Schrettl et al., 2010). SreA is a GATA-type DNA-binding protein that represses iron uptake during sufficiency (Oberegger et al., 2001; Oberegger et al., 2002; Schrettl et al., 2008), while the bZIP protein HapX activates iron uptake and represses iron-consuming processes during iron starvation (Hortschansky et al., 2007; Schrettl et al., 2010; Schrettl and Haas, 2011). Later on, HapX was found to be essential as well in conditions of iron excess, promoting iron storage in vacuoles to prevent toxicity via the transporter CccA (Gsaller et al., 2012; Gsaller et al., 2014). Iron homeostasis in fungi primarily depends on the regulation of iron uptake and storage, as no excretion mechanism for excess iron has been identified. Importantly, a deficiency in HapX, but not SreA, attenuates the virulence of *A. fumigatus* in murine models of aspergillosis. HapX orthologs have also been implicated in virulence in other human pathogens, such as *Candida albicans* and *Cryptococcus neoformans* (Labbe et al., 2007; Haas et al., 2008; Schrettl et al., 2008; Jung et al., 2010; Schrettl et al., 2010; Hsu et al., 2011) or plant pathogens, like *Fusarium oxysporum* (Lopez-Berges et al., 2012).

Additionally, a connection between iron metabolism and hypoxia, another stress condition faced by fungal pathogens during infection, has been identified. In *A. fumigatus*, this link is mediated by the transcription factor SrbA, a sterol regulatory element-binding protein (Willger et al., 2008). Conserved across many eukaryotes, SrbA maintains sterol homeostasis during sterol depletion (Willger et al., 2008; Bien and Espenshade, 2010). It also coordinates sterol biosynthesis, heme biosynthesis, and iron acquisition by regulating reductive iron assimilation (RIA) and siderophore production in species like *A. fumigatus*, *Schizosaccharomyces pombe*, and *C. neoformans* (Willger et al., 2008; Blatzer et al., 2011; Chung et al., 2014; Yap et al., 2023). SrbA’s role in *A. fumigatus* virulence further highlights its importance (Willger et al., 2008; Yap et al., 2023).

In this paper, we describe the development and optimization of two large-scale screening methodologies in *A. fumigatus* to identify genes involved in iron metabolism using large deletion mutant libraries. Our primary objective was not only to identify key players in iron metabolism in *A. fumigatus* but also to create methods capable of processing a large number of strains, which could be applied to other fungal species with limited prior knowledge of their iron metabolism.

## Materials and methods

### Strains and media

The strains used in this study are part of the kinase deletion mutant library created by van Rhijn et al. (2024). In this paper, they are referred to by their code name, which includes plate number, row, and column. A complete list of strains, along with their corresponding gene IDs and, when available, gene name and function, is provided in Supplementary Table S1. Standard growth conditions were set at 37°C using *Aspergillus* minimal medium (AMM) (Pontecorvo et al., 1953), in either solid or liquid form, with 1% glucose as carbon source and 10 mM glutamine as nitrogen source. To induce iron starvation, AMM without iron in the trace elements (−Fe) or AMM supplemented with the iron chelator bathophenanthroline disulfonate (BPS) at a final concentration of 200 μg/mL was used. Iron sufficiency (+Fe) was achieved by adding 30 μM FeSO₄ to the medium, while high iron conditions (hFe) involved either 10 or 20 mM FeSO₄. Plates were incubated for 3 days, or 5 days for slow-growing mutants. Liquid cultures under shaking conditions were grown for 18 h, and growth in 96-well plates was monitored for up to 48 h.

### Spore singling, amplification, collection and counting

Before optimizing the large-scale screening, all strains in the kinase deletion library were first amplified on SAB plates containing 200 μg/mL hygromycin B. Due to the difficulty of achieving true iron starvation, each purified strain was passed twice on AMM −Fe prior to screening. Spores were then collected as required, often from multiple plates, and counted using a hemocytometer. Spores were collected and diluted in a solution of 0.9 M NaCl and 0.1% Tween 80.

### Growth and porphyrin measurements

Mutant strains were inoculated into Nunc96 plates (Thermo Scientific Inc., Waltham, MA, United States) at a final concentration of 1 × 10^5^ spores/mL in AMM, each well containing 100 μL. A CLARIOstar plate reader (BMG Labtech, Ortenberg, Germany) was used to measure optical density at 600 nm (OD_600_) and porphyrin fluorescence (excitation at 397/15 nm, emission at 627/20 nm). To verify that fluorescence measurements correlate with porphyrin fluorescence and optimize the plate reader protocol, protoporphyrin IX (Sigma-Aldrich Corp., St. Louis, MI, United States) was dissolved in Tris-HCl as specific concentration and measured as reference. Plates were also scanned using the IncuCyte S3 Live-Cell Analysis System with a 20× magnification S3/SX1 G/R Optical Module (Essen Bioscience Inc., Ann Arbor, MI, United States). Fungal growth was analyzed using the IncuCyte S3 software’s Basic Analyzer tool (Version 2020; Essen Bioscience Inc.) to measure % confluence. The software-generated confluence mask was exported with each respective image to enhance visualization of cells against the background. Measurements were initially taken every 2 h between 16 and 48 h of incubation at 37°C to determine the optimal endpoint. Based on preliminary results, the final measuring endpoint was set at 40 h.

### Protoporphyrin IX quantification

Protoporphyrin IX (PpIX) levels were quantified using HPLC with UV and fluorescence detection, following the protocol by Bonkovsky et al. (1986), and normalized to protein content in the sample.

### RNA extraction, northern blot and siderophore measurements

For northern blot analysis, *A. fumigatus* strains were grown in 100 mL liquid cultures for 16 h at 37°C in AMM and AMM −Fe. Cultures grown under iron-sufficient conditions served as controls, while iron-starved cultures were used to detect the rapid HapX-induced response, 1 h after the addition of iron at a final concentration of 30 μM (Gsaller et al., 2014). RNA was extracted using TRI Reagent (Sigma-Aldrich Corp., St. Louis, MI, United States) as per the manufacturer’s protocol. For electrophoresis, 10 μg of RNA was separated on a 0.6 M formaldehyde agarose gel, and northern blot analysis was performed using digoxigenin-labeled probes. Band intensities were quantified using *ImageJ* (NIH). Equal-sized rectangular regions were drawn around each band, and the integrated density (area × mean gray value) was measured. Background signal was subtracted using a region adjacent to each lane. The intensity of each target band was first normalized to the housekeeping gene (GAPDH), and values were then scaled relative to the WT strain under control conditions, which was set to 1. Quantification was performed from three independent biological replicates (separately grown cultures processed and blotted independently). Statistical analyses were conducted using an unpaired two-tailed Student’s *t*-test with significant differences indicated by a *p*-value of <0.05.

For siderophore measurements, *A. fumigatus* strains were grown in 100 mL AMM −Fe liquid cultures for 18 h at 37°C. Both extracellular and intracellular siderophores were extracted and quantified according to what previously described by Oberegger et al. (2001). Biomass was freeze-dried and measured for normalization. All experiments were performed in triplicates. Statistical analyses were conducted using an unpaired two-tailed Student’s *t*-test with significant differences indicated by a *p*-value of <0.05.

### Protein extraction and western blot

Protein extraction and western blot analysis were performed following the protocol outlined by López-Berges et al. (2021). In accordance to what previously described, mycelial biomass was first lyophilized and then treated with NAOH and trichloroacetic acid. The western blot nitrocellulose membranes were incubated with anti-HapX antisera (1:20,000; Davids Biotechnologie, Regensburg, Germany) and followed by monoclonal anti-rabbit IgG peroxidase (1:10,000; A1949 Merck KGaA, Darmstadt, Germany) as secondary antibody, for the detection of HapX. Tubulin was used as housekeeping gene for normalization, and this was detected with monoclonal anti-α-tubulin (1:10,000; T6119 Merck KGaA, Darmstadt, Germany) followed by polyclonal anti-mouse IgG peroxidase (1:10,000; A4416 Merck KGaA, Darmstadt, Germany) as secondary antibody. Quantification of protein bands was performed in *ImageJ* as described above. Membranes were either sequentially probed or parallel blots were used to detect HapX and the tubulin control. Signal intensities were normalized to tubulin and the WT under control conditions. Three independent biological replicates were used for quantification.

### Virulence assay

Sixth instar larvae of *Galleria mellonella* (SAGIP, Italy), weighing 0.4–0.5 g, were used for virulence assays. Groups of 20 larvae were injected with 1 × 10^6^ spores in 20 μL of insect physiological saline (IPS) solution into the hemocoel via one of the hind last pro-legs, following the procedure described by Kelly and Kavanagh (2011), and incubated at 37°C. Control groups included untouched larvae and larvae injected with sterile IPS. Survival was monitored every 24 h over a 144-h period, and the experiments were performed at least twice. Statistical analysis was conducted using GraphPad Prism software (vs.9.0.1). Survival curves were compared using the Log-rank (Mantel–Cox) test and the Gehan–Breslow–Wilcoxon test, with significant differences indicated by a *p*-value of <0.05.

### Data processing, graphical representation and statistical analysis

For data visualization, simple plots like bar plots and scatter plots for a reduced number of samples obtained during the development of the screenings were generated in Excel, and significance was calculated with *t*-test (*p*-value <0.05). The results from all large-scale screenings were elaborated for visualization in RStudio 2024.12.0, using the packages ggplot2, ggrepel, tidyr, dplyr, GGally and HTSvis. Pairwise Pearson correlation coefficients were calculated in R using the cor () function and data were visualized using scatter plots and pairwise plot matrices, where correlation coefficients are shown in the upper panels. A positive coefficient close to 1 indicates a strong positive linear correlation, while values near 0 suggest weak or no correlation.

## Results

### Development of low iron-adaptation screening methods

The most common strategies for the study of iron related phenotypes involve spot assays onto solid agar medium with different iron concentrations, as well as growth of liquid cultures for biomass and siderophore measurements. The challenge of screening mutant libraries for impairments in iron metabolism is essentially related to how the above-described methodologies can be upscaled to be high throughput, as both liquid cultures and spot assays for hundreds of strains would require a considerable amount of work, time, and materials. In an attempt to develop new strategies, we first selected *A. fumigatus* as key organism, due to the extensive work already performed in the characterization of iron metabolism in this particular species. In *A. fumigatus* there are already several genes known to be pivotal in the regulation of iron sensing and uptake, as well as in the process of iron metabolization and detoxification. We decided to use two specific mutant strains as control to develop and tune our screenings, each strain displaying specific phenotype if subjected to various iron concentration in their environment: ∆*hapX* and ∆*srbA*.

**Figure 1:**
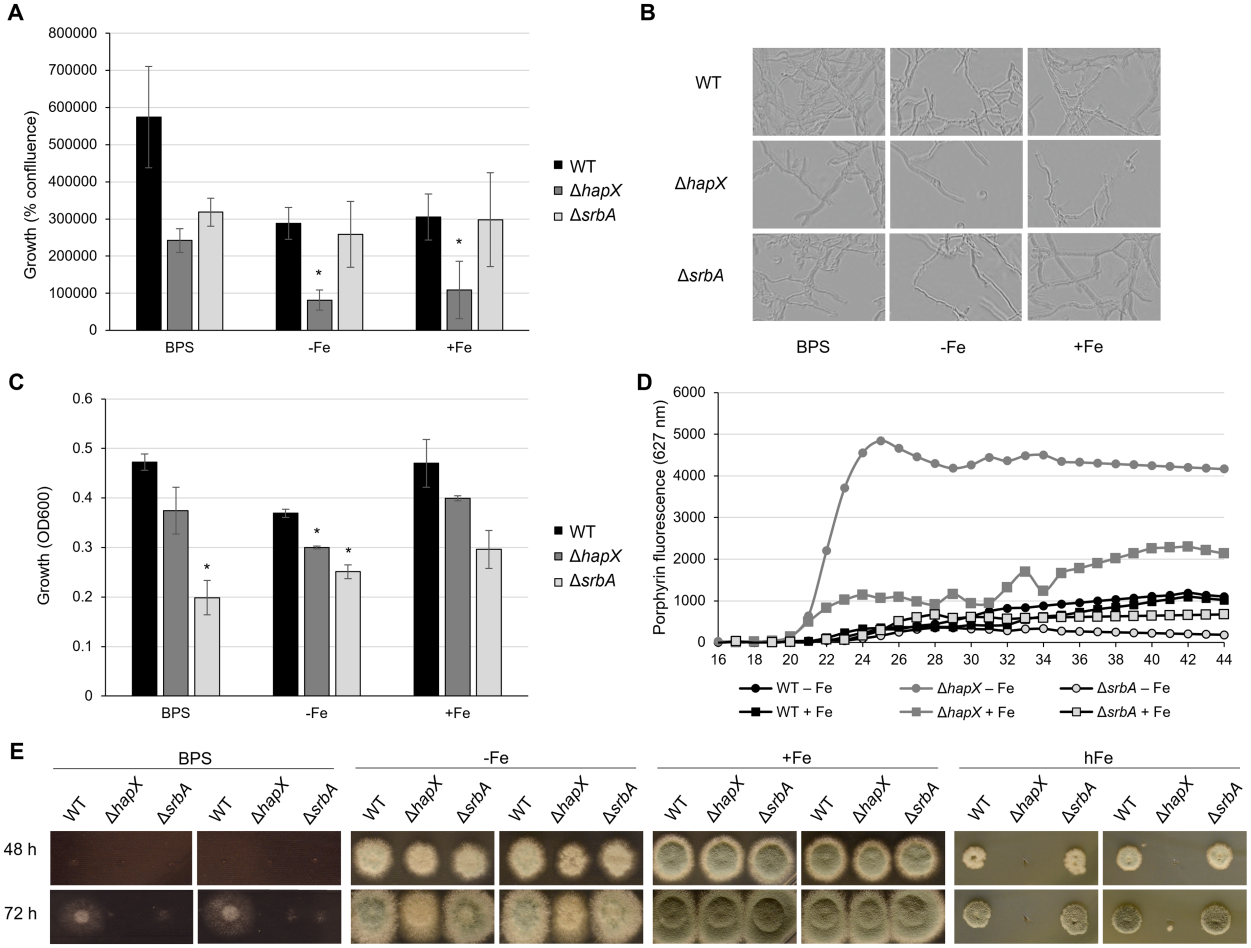
Development of screening methodology for iron-related defect suitable for upscaling. Results of preliminary assays aiming to identify iron-related phenotypes using Δ*hapX* and Δ*srbA* as test subjects in comparison to the WT. **(A)** Measurements of iron-dependent hyphal confluence in 96-well plates obtained with Incucyte S3 after 40 h incubation at 37°C. Significant differences, indicated in the figure with an asterisk, were calculated with Student’s *t*-test (*p* < 0.05). **(B)** Representative examples of Incucyte S3 images used to determine confluence measurements. **(C)** OD_600_ measurements from 96-well plates incubated for 40 h at 37°C in different iron conditions. **(D)** Time course measurements of porphyrin fluorescence taken every 2 h after 16 h preincubation at 37°C. **(E)** Spot assay on solid medium testing reduced-volume inoculum and narrow distance between colonies.

As a first strategy, we tried to substitute the liquid culture setup from shaking flasks to static 96-well plates. ∆*hapX* and ∆*srbA* were inoculated in replicates in a microtiter plate, together with the WT, to monitor their growth over time by measuring the optical density as OD_600_. Measurements were taken every 2 h, starting after 16 h pre-incubation at 37°C, and continuing until 48 h. Previous studies reported that OD_600_ measurements for filamentous fungi are not always considered reliable, particularly when not keeping into consideration variables like the morphology changes (Hameed et al., 2024). We decided therefore to employ as monitoring/confirming analysis measurements of hyphal confluence, obtained via Incucyte S3, as well as visual evaluation of each well. This setup with liquid culture in 96-well plates was immediately found not suitable to analyze conditions involving high concentrations of iron, like 10 mM, as the iron precipitation and the consequent turbidity in each well did not enable correct readings by the instruments nor visual evaluation of growth. Hence, the only condition that could be tested was iron starvation, either in AMM −Fe or with addition of BPS, in comparison to iron availability, in AMM containing iron at the final concentration of 30 μM (+Fe). Confluence measurements were able to discern differences in growth between the three strains following 20 h incubation in iron starvation and iron sufficiency conditions, however these differences were more prominent after 24 h. At later time points, hyphal biomass, particularly in iron sufficiency, saturated the instrument’s ability to accurately measure growth. Interestingly, in contrast to our previously published data, we did not detect any growth defect of ∆*srbA* in −Fe nor in the control conditions when compared to the isogenic isolate (WT) (Figures 1A,B) (Schrettl et al., 2010; Blatzer et al., 2011). Moreover, in contrast to our expectations, the WT and ∆*hapX* isolates exhibited improved growth in the presence of BPS compared to iron replete conditions. We therefore considered this strategy to be sub-optimal for the detection of strains that exhibit defects in the presence of BPS.

As quantification via Incucyte was not possible after 24 h, we instead assessed optical density measurements (OD_600_). Up to 34 h of incubation, OD_600_ measurements did not show a robust difference between inoculated and non-inoculated wells. At later time points OD_600_ measurements were in agreement with visual evaluation of growth, and 40 h of incubation was estimated as the best time point with respect to consistency within replicates (Figure 1C). However, the setup of liquid culture in 96-well plates was confirmed again as not suitable to study growth in the presence of BPS, as both WT and ∆*hapX* showed no significant difference in comparison to the control condition. Nevertheless, this strategy evidenced a significant growth defect of both ∆*hapX* and ∆*srbA* in comparison to the WT in −Fe, while in normal iron no significant impairment was detected (Figure 1C).

A second phenotypic strategy for detecting defects in iron-homeostasis is to screen using porphyrin fluorescence. Porphyrins, like PpIX, are characterized by the ability to generate autofluorescence by excitation at 397 nm (Hortschansky et al., 2007; Schrettl et al., 2010). In iron sufficient conditions, iron would be regularly incorporated into PpIX forming heme, which quenches fluorescence. To avoid accumulation of toxic PpIX during iron starvation, the heme biosynthetic pathway is downregulated via HapX (Oberegger et al., 2002; Hortschansky et al., 2007). We aimed our screening to the identification of genes affecting directly or indirectly HapX, based on the fact that ∆*hapX* showed PpIX accumulation during iron starvation. Due to our previous results, we decided to exclude the BPS condition from this screening and to focus only on the two condition of iron starvation (−Fe) and iron sufficiency (+Fe). Strains were pre-incubated at 37°C for 16 h, and increases in PpIX fluorescence was monitored for an additional 28 h (Figure 1D). Consistent differences between strains and conditions became visible after 22 h, but the fluorescence level plateaued for most strain by 38 h (Figure 1D). As expected, D*hapX* showed a strong increase in porphyrin fluorescence in comparison to the WT in −Fe and, interestingly, a higher accumulation of porphyrins was even detected in iron replete conditions (+Fe). The fluorescence generated by D*srbA* was reduced compared to the wild-type in +Fe indicating lower porphyrin accumulation, and this reduction was even more prominent in −Fe. An important fact to consider, at this point, was that these differences in porphyrin accumulation did not take into account the growth differences between strains. We selected therefore the 40-h time point, which would allow normalization of the fluorescence to the fungal growth, to screen the mutant library for defects in PpIX accumulation.

In summary, for further screening purposes, the methodologies of choice to be conducted on 96-well plates were: (i) growth evaluation via OD_600_ measurements and (ii) porphyrin fluorescence measurements, both taken after 40 h incubation for the condition of −Fe and +Fe. Confluence measurements were not found suitable due to the instrument limitation after 24 h incubation (lack of focus in the images and difficulties in calculating confluence on a three-dimensional growth system). The condition of iron starvation including BPS was excluded due to the inconsistent results obtained for this specific set up in 96-well static culture, and especially considering the discrepancies emerged with previously published results on solid media.

### Development of screening methods for high iron-sensitivity

The major limitation of our new screening strategies remained the inability to screen for high iron sensitivity, therefore we thought of a way to adapt the usual assay for growth assessment on solid media to a large-scale setting. The idea behind this method was to identify a specific time, iron concentration, distance between colony inoculation, concentration and volume of the inoculum, and other possible parameters that would allow the use of a multichannel pipette and the visualization of spot assay differences in a limited space. WT, ∆*hapX* and ∆*srbA* were again selected as control strains for preliminary tests. We also conducted the assay in iron starvation as apparent phenotypes of mutants affected by iron starvation could be detected in previous work. After 24 h, colony growth was not sufficient to allow any differentiation between strains, irrespective of the inoculum volume. A 5 μL inoculum containing 10^4^ spores (2 × 10^5^ spores/mL), spotted (9 mm apart) with a standard 12 channel multipipette, resulted in colonies that after 48 h at 37°C were already overlapping with each other in −Fe and + Fe condition (data not shown). To avoid early contact between different forming colonies, we reduced the volume for point inoculation to 3 μL (6 × 10^2^ spores) (Figure 1E). Plates containing BPS were found not suitable for this type of screening, presenting no growth with the standard inoculum for the first 48 h, while later on showing thin hyphal growth with larger radius than seen in −Fe. In −Fe, instead, both Δ*hapX* and Δ*srbA* showed only a very slight growth reduction, that did not align well with the intended purpose to select for strains with defect under iron deficiency in a large screening. For the high iron condition (hFe), a final concentration of 10 mM was initially tested, but did not show growth differences to the expected extent (data not shown). Hence, for final optimization, we tested 20 mM iron with the reduced inoculum, and with this setup we were able to reproduce the strong growth defect of ∆*hapX* in comparison to both WT and ∆*srbA* (Figure 1E) (Gsaller et al., 2014).

### Upscaling and validation of the developed screening strategies

**Figure 2:**
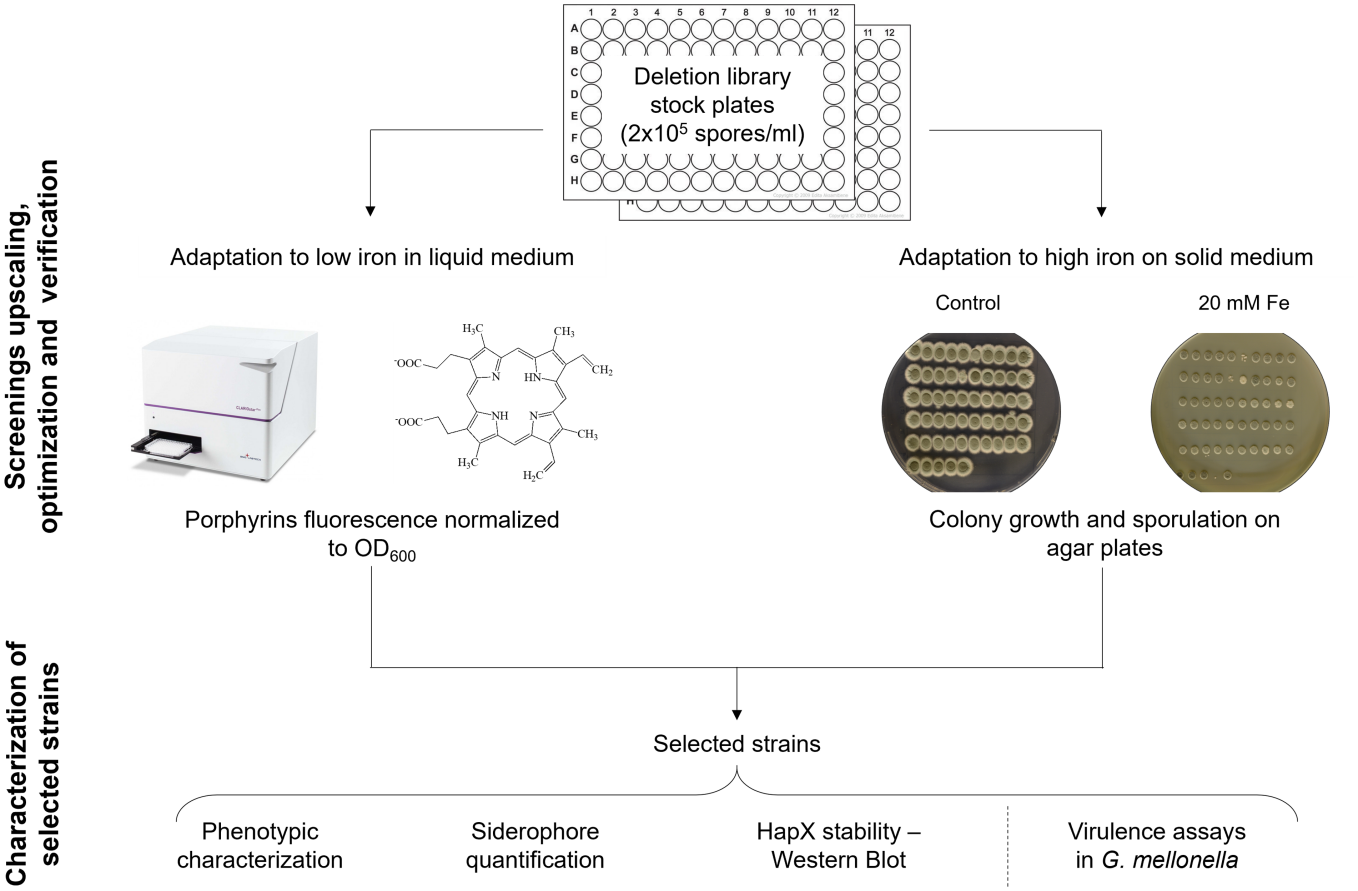
Screening upscaling and verification strategy. Schematic workflow followed for the upscaling and validation of the developed screening methods, followed by a phase of analysis to establish if one of them could be preferable over the other via various iron specific assays on a subset of strains.

Before starting the screening of the protein kinase (PK) mutant library (van Rhijn et al., 2024), spores of each strain were diluted in spore buffer to a final concentration of 2 × 10^5^ spores/mL in 96-well plates, to have stock plates already organized and arranged to perform inoculation for all further assays. Unfortunately, any attempt to add small percentage of glycerol and store the plates at −80°C for long term conservation resulted in decreased fitness for many strains and in an inconsistent manner. However, we found that storing the stock plates for few days at 4°C did not affect fungal growth or the outcome of the screening. The pipeline that we decided to follow is summarized in Figure 2.

**Figure 3:**
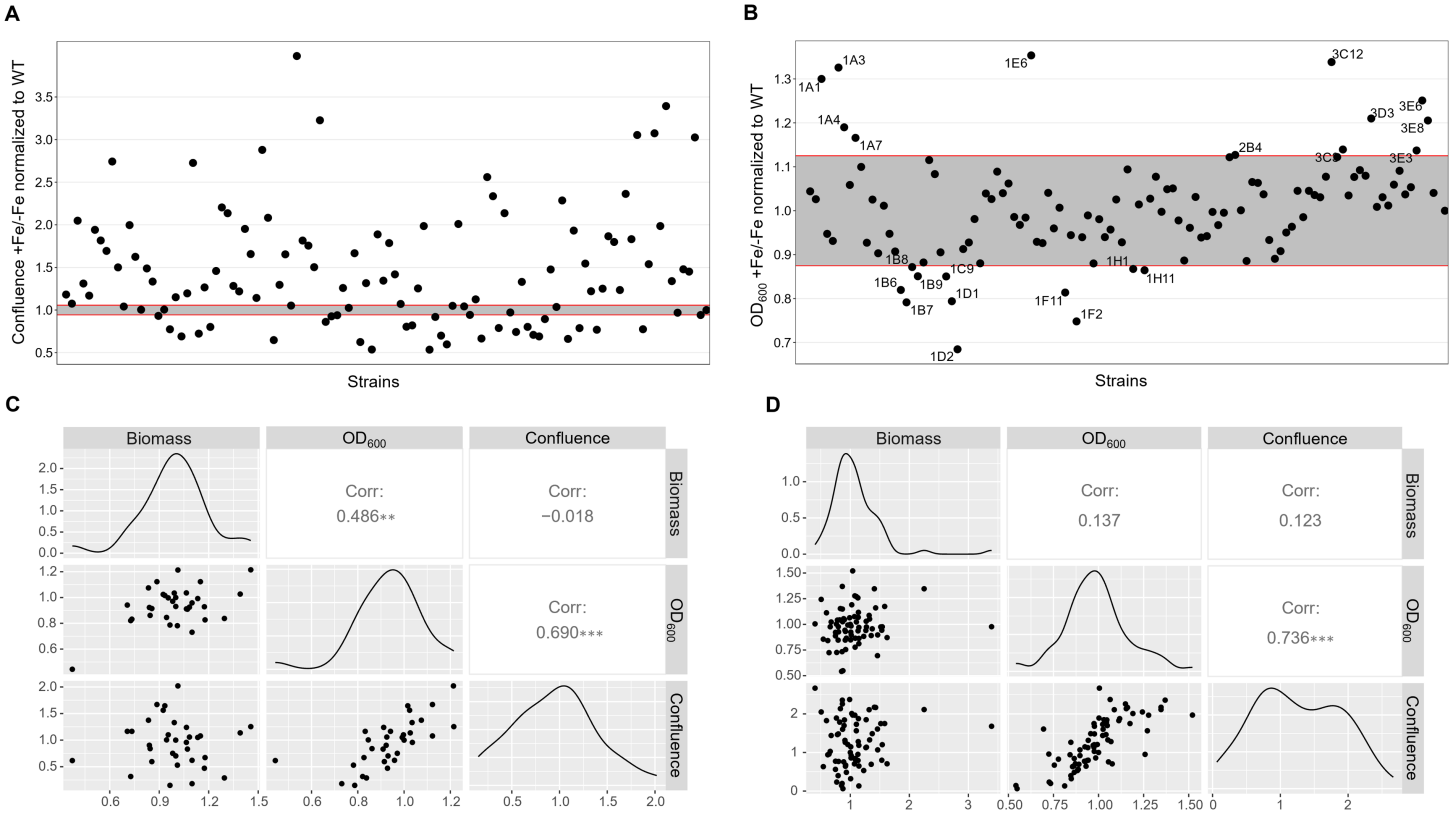
Low iron screenings in static liquid culture. Scatter plot of +Fe/−Fe growth measurements obtained with quantification of confluence **(A)** or OD_600_ **(B)** of the protein kinase deletion mutant library in comparison to the WT and the control Δ*hapX* and Δ*srbA*. Pairwise scatter plot matrix with correlation coefficients calculated between confluence measurements, OD_600_ and biomass quantification after growth in liquid shaking culture for a randomly selected number of strains in −Fe **(C)** and +Fe **(D)** conditions.

To first assess the growth behavior in the respective conditions, the 109 strains available from the PKs library were inoculated in 96 well plates for confluence and OD_600_ measurements after 40 h incubation at 37°C in AMM either without or with iron. ∆*hapX* and ∆*srbA* were included as control, but not used to set a defined threshold. We wanted to develop methods that could be applied to many different species, where perhaps no preliminary knowledge about mutant strains with significant phenotype in diverse iron concentration is available. Therefore, the threshold to identify significantly different strains was established by using the variation between multiple measurements just for the WT. It was immediately clear that confluence measurements for the WT were extremely consistent, making this variability interval for WT-like growth very small (Figure 3A). To simplify the visualization and evaluation of the screening outcome, we decided to focus on the comparison between growth in normal iron versus growth in iron starvation, provided as ratio between confluence measurements in the two conditions for each strain (confluence in +Fe/confluence in −Fe). Too many strains showed positive ratios, reflecting reduced growth in −Fe in comparison to the WT, to consider this method as a valid screening. In contrast, the same data analysis strategy applied to the OD_600_ measurements identified only 23 strains as significantly different from the WT, 12 showing reduced growth and 11 increased growth in −Fe (Figure 3B). Unfortunately, with this new evaluation method both ∆*hapX* and ∆*srbA* were not identified anymore, due to slight growth defects measured in +Fe as well, implying this screening strategy and, particularly the data elaboration needed for large number of strains, are not suitable to identify iron related phenotypes with adequate sensitivity.

**Figure 4:**
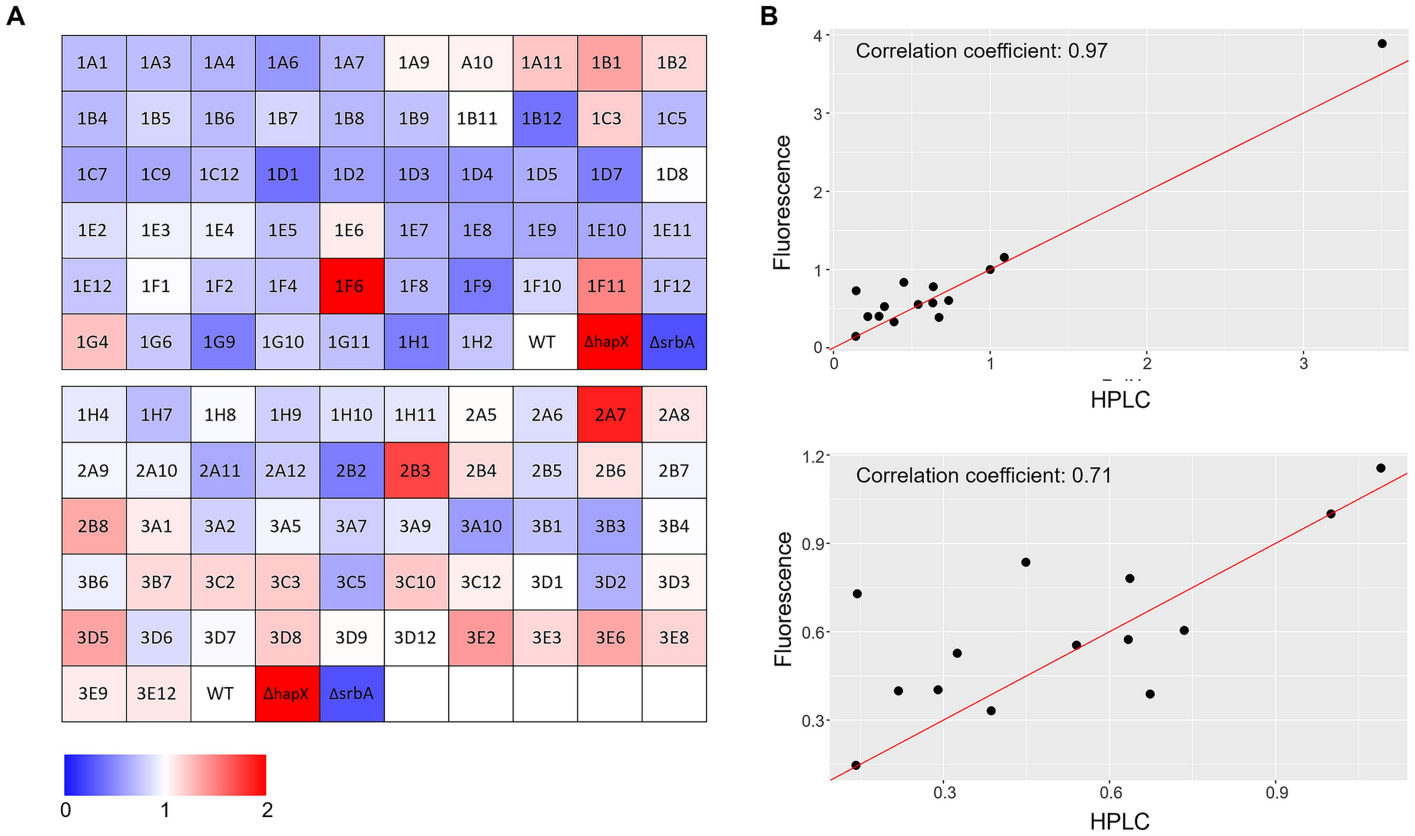
Porphyrin-fluorescence measurements. **(A)** Color coded visualization of the two 96-well plates used for porphyrin-fluorescence measurements in −Fe normalized over values obtained in +Fe for the protein kinase deletion mutant library in comparison to the WT and the control Δ*hapX* and Δ*srbA*. **(B)** Scatter plot with correlation coefficients calculated between fluorescence measurements and specific PpIX quantification via HPLC for a randomly selected number of strains including Δ*hapX* (top panel) or not (bottom panel).

Correlation with liquid shaking culture was performed for verification by growing randomly selected strains (among both screening-identified strains and not) in liquid shaking culture, in −Fe and +Fe, and measuring the lyophilized biomass (Figures 3C,D). We could then verify that confluence and OD_600_ measurements showed highly significant correlation between them in either condition (Corr. 0.690 and 0.736 in −Fe and +Fe, respectively). Dry weight measurements, instead, were not significantly correlating with the confluence values (Corr. −0.018 and 0.123 in −Fe and +Fe, respectively) but significantly correlating with OD_600_ (Corr. 0.486) in −Fe. This data indicated that, albeit OD_600_ measurements could not be used directly as large-scale screening for impairment to adaptation to iron excess, at least they could be used as indication of fungal growth in −Fe in a reliable way. Simultaneously with the OD_600_ measurements, porphyrin fluorescence evaluation at 397 nm was recorded. For an easier evaluation of the outcome, we first calculated the ratio between the two conditions and then normalized the obtained values to the WT (Figure 4A). To maintain the possibility to use this screening method in a broad range of species, the threshold to identify significantly different strains was again established as standard deviation calculated solely on multiple measurements for the WT. According to these parameters, we were able to select 54 strains, of which half accumulated more porphyrins than the WT and half less. In this case, to validate the efficiency of the screening PpIX was extracted from the freeze-dried mycelia of those strain randomly selected in the previous step to assess biomass formation (Figure 4B). PpIX HPLC measurements mostly confirmed the results obtained with the plate reader screening, with a Pearson correlation coefficient of 0.97 (0.71 when excluding D*hapX* in the correlation analysis), proving the validity of porphyrin quantification in 96 well plates and suggesting that PpIX could be the main accumulated porphyrin that is then measured in the plate reader.

**Figure 5:**
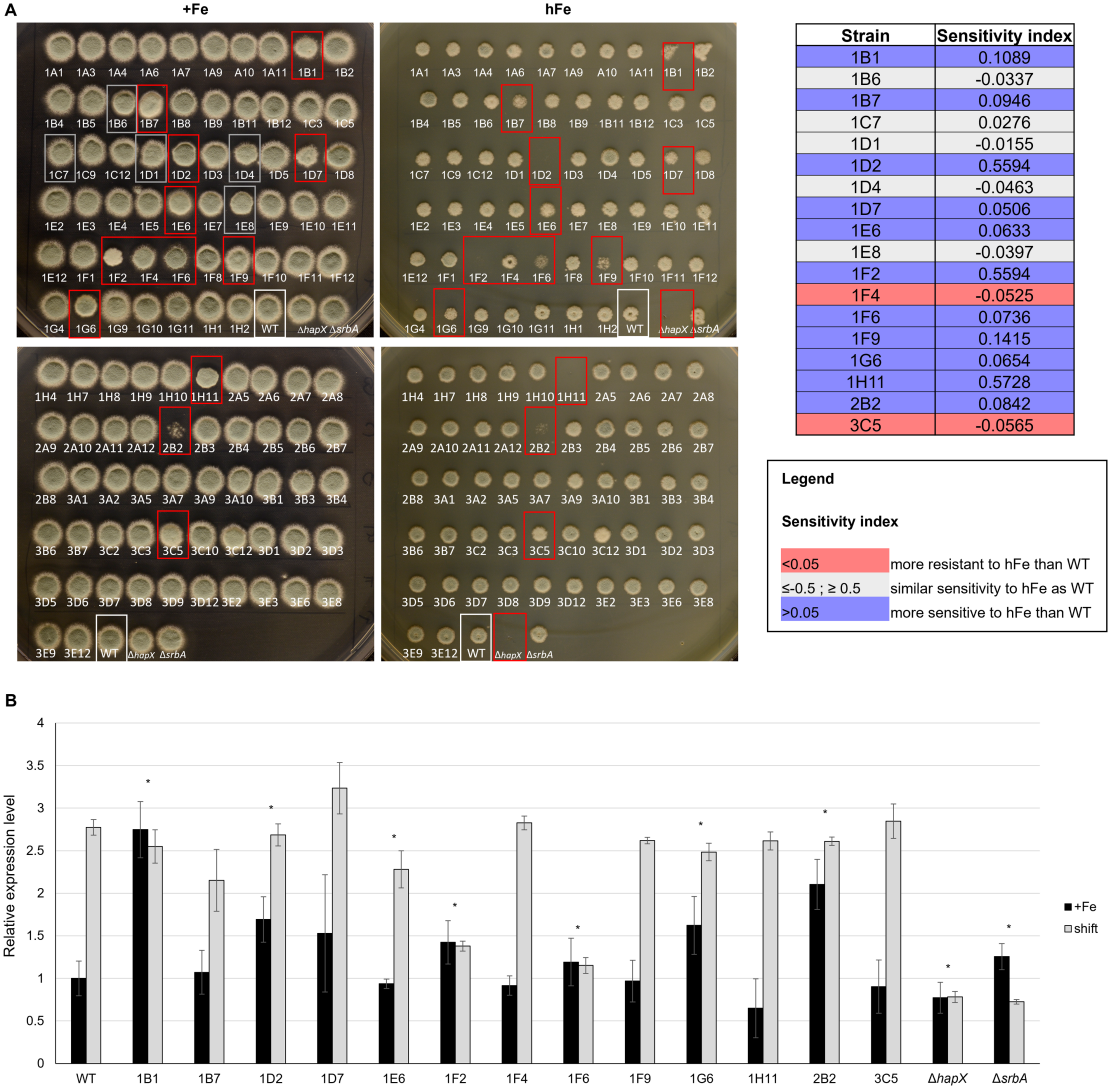
High iron screening on solid medium. **(A)** Spot assay with reduced volume and distance of inoculum to assess high iron-related phenotype among strains in the protein kinase deletion mutant library in comparison to the WT and the controls Δ*hapX* and Δ*srbA*. Confirmation of visual evaluation is indicated for the identified strains in the table on the right as sensitivity index. **(B)** Northern blot evaluating expression level of the vacuolar iron importer *cccA* in the strains identified with the high iron screening in standard medium (+Fe) and in a shift condition, i.e.1 h after adding Fe to iron starved-cultures. Signals were normalized to the WT in +Fe and significance, indicated in the figure with an asterisk, was calculated with Student’s *t*-test (*p* < 0.05).

For the high iron screening, 13.5 cm petri dished were prepared with control medium, containing 30 μM of FeSO_4_, or high iron medium, with a final concentration of 20 mM. Strain order and arrangement were consistent with those used to inoculate a 96-well plate, so 3 μL from the dilutions prepared for the previous assays could serve as inoculum. Evaluation of colony growth was carried out after 48 h of incubation at 37°C (Figure 5A). Excluding D*hapX* and D*srbA*, 13 strains showed an altered hFe susceptibility compared to WT, keeping into account their respective growth in +Fe (Figure 5A, highlighted in red). Five strains were found to have a consistent growth defect (*n* = 3) when compared to the WT in both hFe and normal iron concentration, hence they were considered to have an iron-independent growth defect (Figure 5A, highlighted in grey). Verification of visual evaluation was performed by measuring the diameter of each selected colony and using the average measurement to calculate a sensitivity index to hFe in comparison to the WT. This index was calculated subtracting the ratio of growth in hFe over the control condition (+Fe) for the mutant from the same ratio for the WT. A positive value for the sensitivity index indicated mutant strains with increased sensitivity to hFe in comparison to the WT, while a negative value suggested reduced sensitivity. To further explore the growth differences in these hits, each strain was spot inoculated on agar to enable assessment of growth for a longer period of time (Supplementary Figure S1). In this verification, to adhere to our standard characterization conditions, the concentration of iron for hFe plates was decreased to 10 mM, and iron starvation with or without BPS was also included in the assay. The phenotypes identified with the screening were mostly confirmed, despite particularly for the hFe condition they were found less accentuated (likely due to the decreased concentration of iron). The few exceptions were represented by 1D4 (uncharacterized serine/threonine kinase showing high similarity to *S. cerevisiae* Sat4), identified with a growth defect in the plate screening in +Fe but no defect in the confirmation plates, and the two strains 1A10 (uncharacterized serine/threonine kinase showing high similarity to *S. pombe* Cek1) and 1E12 (uncharacterized serine/threonine kinase showing low similarity to kinases from *S. cerevisiae* or *S. pombe*), not identified in the screening but exhibiting reduced growth during the verification process.

To exclude a general sensitivity to high metal concentration, rather than to iron specifically, strains that showed highly different phenotype from the WT in the high iron screening were tested in the presence of 2 mM copper (hCu) or 10 mM zinc (hZn) (Supplementary Figure S2). No significant impairment was found in the presence of high concentration of the other two metals, implying specificity for iron. As intracellular storage of iron and iron detoxification generally occur via CccA-mediated vacuolar iron deposition, these strains were also analyzed for different expression of *cccA* via Northern blot (Figure 5B). As expected, the WT exhibited an upregulation of *cccA* expression when facing a sudden higher concentration of iron in the surrounding environment after a period of iron starvation (shift) in comparison to the normal iron level present during cultivation in iron sufficiency. Of the selected strains presenting a phenotype in the high iron screening, some presented only difference in *cccA* expression level during iron sufficiency cultures, but not in the shift condition, e.g., 1B1 (Δ*ste7*), 1D2 (Δ*bck1*) and 2B2 (Δ*uvsB*). More than half of them, however, presented altered level of *cccA* in the shift set up, 1D7 (Δ*sskB*) with an increased expression and the other five with lower expression, more or less accentuated but significant. Surprisingly, some of the strains that exhibited the strongest growth defect in high iron plates, like 1F9 (Δ*ppd7*) or 1H11 (Δ*mpkA*), did not present any defect in the expression of *cccA*, suggesting the impairment in a different mechanism for iron detoxification.

### Evaluation of the screening methods efficacy in selecting relevant strains

**Figure 6:**
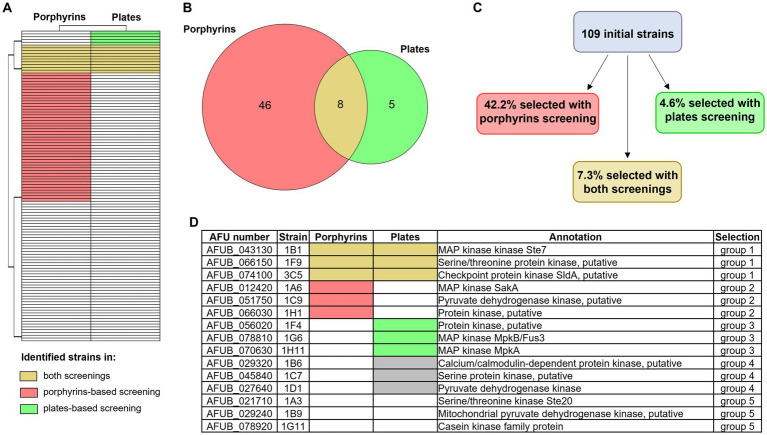
Overview of the two newly developed screening results. Heatmap **(A)** and Venn diagram **(B)** showing the number of strains of the protein kinase deletion mutant library that were identified as significantly different from the WT with the porphyrin fluorescence-based screening (in pink) or the high iron plates (in green). Strains that were identified with both screening are highlighted in yellow. **(C)** Percentage of strains that were selected with one of the other screening (porphyrin-based in pink and high iron screening in green), or with both of them (in yellow). **(D)** Table summarizing which strains were selected for further investigation, in order to evaluate the potential of each screening and their efficacy. Group 1 included strains selected with both screening methods, group 2 those selected via the porphyrin-based one and group 3 those selected via the high iron screening. Groups 4 and 5 represent the control groups: the first one including strains that showed reduced growth in the control condition (+Fe) and the second strains that showed no altered phenotype in comparison to the WT.

The results of the above-described large-scale screenings are summarized in Figures 6A–C and in the Supplementary Table S2. Eight strains were selected in both screening methods, three of which (1B1/∆*ste7*, 1D2/∆*bck1* and 1D7/∆*sskB*) having deleted one of the mitogen activated protein kinases (MAPKs) pathway encoding genes. Notably, 1B1 showing increased signal while 1D2 and 1D7 reduced fluorescence in the porphyrin screening. More strains lacking one of the MAPKs genes were identified only in one of the screening approaches, one presenting lower level of porphyrins in the fluorescence-based screening (1A6/∆*sakA*) and two exhibiting reduced growth in the high iron screening (1G6/∆*mpkB* and 1H11/∆*mpkA*), corroborating the already reported connection between these signaling pathways and iron metabolism (Jain et al., 2011; Kaba et al., 2013; Pujol-Carrion et al., 2021). To verify if both screenings are needed for a more precise identification of interesting strains or if one screening alone could be sufficient, we decided to conduct a more extensive characterization on a restricted number of strains (Figure 6D). We selected three strains identified in both methods (group 1) and compared them with three strains retrieved exclusively in one of the screenings (groups 2 and 3 for the porphyrin fluorescence-based screening and the high iron plate screening, respectively). As control, we decided to include three strains that showed impaired growth not only in the presence of high iron but already in standard condition of iron sufficiency, to exclude general effects directly linked to poor growth and not necessarily connected to iron metabolism (group 4), as well as three strains that exhibited growth comparable to the WT and were not selected in any screening (group 5). For each experiment, WT, D*hapX*, and D*srbA* were also included.

First of all, we repeated a growth assay on solid medium, comparing the effect of different iron concentrations in standard growth condition in combination with other stress factors, such as hypoxia, light and lower or higher temperature (Supplementary Figure S3). All tested strains showed various growth alterations in comparison to the WT, without clear consistence within groups. Strains in group 1, which were selected in both screenings, showed more similarities with strains in groups 3 and 4, selected in the high iron plate assay or with poor growth also in iron sufficiency. However, these growth defects appeared consistent not only in the condition of normoxia, but were reproduced for almost all iron concentration tested in hypoxia, growth in presence of light, at 25°C and at 48°C (with the exception of the putative kinase 1F4 from group 3 and 1C7 and 1D1 from group 4 that showed better growth with light). Strains selected with the porphyrin screening (group 2), instead, were characterized by a rather similar growth to the WT, often with some coloration differences, and appeared more affected by temperature (decreased growth at 48°C for 1A6/SakA and 1C9, a putative pyruvate dehydrogenase, while better growth for 1H1, a putative kinase) than by different iron concentrations. Stronger growth defects were found for 3C5 (Δ*sldA*), 1G6 (Δ*mpkB*) and 1B6 (a putative calcium/calmodulin-dependent protein kinase) in −Fe, 1B1 and 1H11 (Δ*ste7* and Δ*mpkA*, respectively) in hFe at 25°C, as well as 1B1 (Δ*ste7*), 1F9 (Δ*pdd7*), 1C9 and 1B9 (two putative pyruvate dehydrogenase kinase) in general at 48°C. It is important to highlight that some phenotypic discrepancies between these phenotypic results and the high iron-plate screening, for example the strains 1B1 and 1F4 not showing any more a growth defect in hFe, could be explained with differences in the experimental setup, like the final iron concentration or inoculum size. Similarly, in this last characterization all strains in group 1 and 1H11 were characterized by a slight growth decrease in normal iron condition, again possibly attributable to the different experimental setup. Specifically in regard to strain 1H11, Δ*mkA* in *A. fumigatus* is already known to exhibit a general growth defect in comparison to the WT (Valiante et al., 2009; Alves de Castro et al., 2024), and such defect seems to be aggravated in hFe.

**Figure 7:**
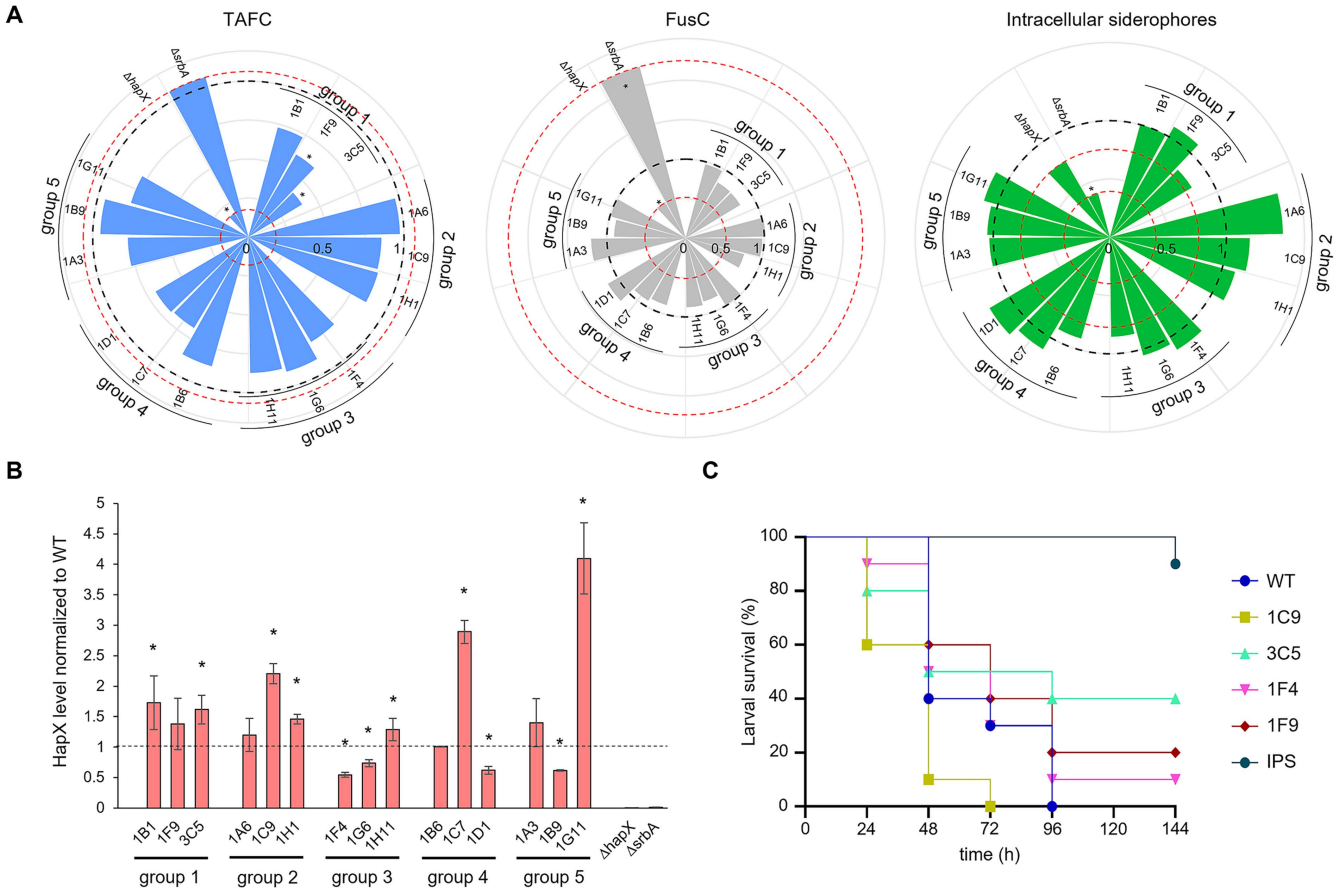
Iron targeted characterization of selected strains. **(A)** Siderophore quantification after growth in liquid culture. TAFC production is represented in light blue on the left plot, FusC in grey in the middle plot and intracellular siderophore in green on the right one. The black dashed line is fixed at value of 1 and represent the WT. The red dotted lines are in correspondence of the relative values for the control strains Δ*hapX* and Δ*srbA*. Significant differences from the WT (*p* < 0.05) are indicated by an asterisk (*). **(B)** HapX protein level detected via western blot normalized to the WT as well as to the housekeeping gene. The dashed line is provided to facilitate visualization and correspond to the WT expression level. The asterisks are indicating strains with a significantly altered HapX level. **(C)** Virulence assay performed in *G. mellonella*. As in the preliminary test only few strains seemed to induce a different outcome compared to the WT, to minimize the number of subjects used only a sub selection was chosen to repeat the assay. IPS injections were used for the control group.

We continued characterization of strains by investigating siderophore production (Figure 7A). Extraction and quantification of both extracellular, triacetylfusarinine C (TAFC) and fusarinine C (FusC) and intracellular siderophores from liquid culture was performed. Strains belonging to group 1 and group 4 showed the lowest production of TAFC, which in the case of group 4 (strains growing less than the WT even in standard condition) could be expected due to the lower biomass production and the corresponding lower consumption of residual iron in the medium (Figure 7A, left plot). Strains in group 2 and 3, instead, had a rather similar extracellular siderophore production compared with WT, with the exception of 1F4 for TAFC and 1H1 for FusC (both uncharacterized serine/threonine kinase showing low similarity to kinases from *S. cerevisiae* or *S. pombe*) (Figure 7A, left and middle plots). For the production of intracellular siderophores, instead, no clear pattern was observed between groups, and the differences to the WT were considered negligible with the exception of 1A6 (Δ*sakA*) showing higher production (Figure 7A, right plot). In regard to statistically significant differences, only group 1 appeared relevant, with 1F9 and 3C5 (Δ*pdd7* and Δ*sldA*, respectively) showing a *p*-value of <0.05 for TAFC production.

To see if a direct connection with HapX phosphorylation and, consequently, its stability, could be associated to any particular group of identified strains, we performed western blot analysis using an anti-HapX antibody (López-Berges et al., 2021). Again, no correlation could be identified within groups, as two strains out of three generated a higher level of HapX in group 1 and 2, two strains from group 2 were found with lower HapX levels and one strain instead higher, and for groups 4 and 5 one strain induced higher HapX and a second again lower (Figure 7B).

Lastly, to verify if perhaps the screenings could be effective in identifying strains with reduced virulence, we performed *G. mellonella* infection assays. After an initial pre-assay, in an attempt to reduce the number of subjects for infection (data not shown), only few strains showed somehow different outcome from that of the WT. These strains included 1F9 and 3C5 (Δ*pdd7* and Δ*sldA*, respectively), which were selected by both screenings, 1C9 (putative pyruvate dehydrogenase kinase), identified only by porphyrin screening and 1F4 (uncharacterized), selected only by high iron-plates assay. The infection assay was repeated only for these four strains and the WT (Figure 7C). Even if no significance was found, perhaps due to the reduced number of larvae imposed by our purely explorative intent, 1C9 appeared even more aggressive towards *G. mellonella* larvae than the WT, while 1F4 produced a minimal increase in larvae survival. The two strains identified with both screening methods showed a consistent tendency to reduced virulence, with 1F9 having only a small improve on survival rate and 3C5 a clearer one.

**Figure 8:**
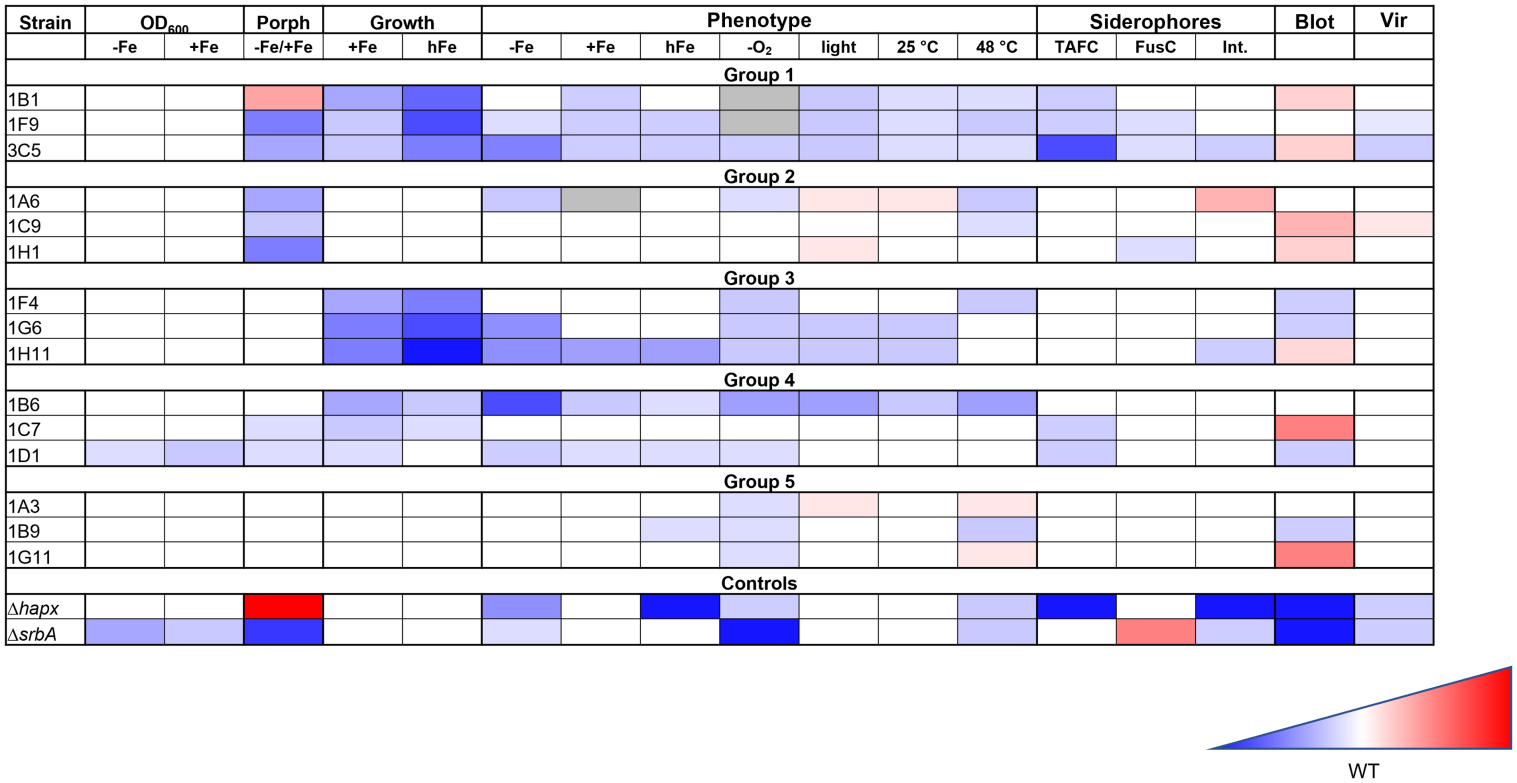
Summary of the iron targeted characterization results in association to the method used for the strain identification. Gradient color-coded summary to verify possible association between screening methods and characterization results. Blue is associated to reduced growth or lower level, while red to increased growth of higher level. White is considered a similar growth level to the WT. In the summary results for the first two methodologies tested are included, based on growth evaluation via confluence quantification or OD_600_ measurements, as well as those for the porphyrin-fluorescence screening (Porph) and the high iron plates screening (Growth). Results for the phenotypic characterization on plates are simplified, not showing all different iron conditions for each stress factor, but only the general effect that was considered due to the stress factor itself. Results for the siderophores measurements, the HapX level (Blot) and the virulence assay (Vir) are also included.

For clearer visualization, all results have been summarized in a color-coded way in Figure 8.

Considering all the results from different experiments and characterization, despite no clear pattern could be identified in the strains selected with one methodology or the other, there seem to be a strong indication that both screening strategies are effective in identifying strains with iron-related defects. The porphyrin-based screening identified a larger number of strains, while the high-iron plate screening appeared more stringent. Strains with stronger defects were not always, but often identified with both strategies, so a combination of both screenings might lead to a higher chance of finding strains with lower virulence potential.

## Discussion

Iron is an essential element for many biological processes, including respiration, DNA synthesis, and cellular metabolism (Ramos-Alonso et al., 2020). However, due to its limited solubility and potential to generate harmful reactive oxygen species, its homeostasis must be tightly regulated across all organisms (Misslinger et al., 2021). Given iron’s pivotal role in the survival and adaptation of pathogenic species within their hosts (Philpott, 2006; Haas, 2012; Martinez-Pastor and Puig, 2020; Stanford and Voigt, 2020; Misslinger et al., 2021), understanding the molecular mechanisms governing iron metabolism and figuring out which players are involved is crucial for identifying novel therapeutic targets.

Here, we present two complementary screening approaches to identify genes involved in iron metabolism from existing deletion mutant libraries, using *A. fumigatus* as a model. The central role of iron in *A. fumigatus* pathogenicity (Haas et al., 1999; Haas, 2003; Schrettl et al., 2004; Hortschansky et al., 2007; Schrettl et al., 2008; Schrettl et al., 2010; Blatzer et al., 2011; Schrettl and Haas, 2011; Gsaller et al., 2012; Haas, 2012; Gsaller et al., 2014; Misslinger et al., 2018; López-Berges et al., 2021; Happacher et al., 2023; Yap et al., 2023) highlights the potential of genes involved in iron metabolism as prime targets for new antifungal treatments. However, the question of how to identify iron-related genes in species where iron metabolism is less understood remains unanswered. Our strategies have been tested on a deletion mutant library generated in *A. fumigatus* (van Rhijn et al., 2024) to evaluate how many already known genes related to iron metabolism could be identified. While the total number of mutants screened was modest (109 strains from a kinase library), the depth and scope of phenotypic validation allowed us to benchmark the approaches and assess their potential scalability.

The first strategy—porphyrin fluorescence normalized to OD_600_ under iron-starved conditions—proved effective in detecting mutants with disrupted intracellular iron balance, as reflected by altered PpIX levels. Despite being able to only measure fluorescence at specific wavelengths and associate it to fluorescence generated by porphyrins in general, this assay correlated well with HPLC-validated PpIX quantification and identified numerous kinases already implicated in iron homeostasis, such as components of the MAPK pathway (e.g., SakA, Ste7, Bck1, SskB) supporting the biological relevance of the readout (Jain et al., 2011; Gsaller et al., 2014) and many other so far uncharacterized proteins.

Growth in standing liquid culture, however, had the major limitation of not allowing screening for hFe related defects, due to high occurrence of precipitates that interfered with growth quantifications. In contrast, the second approach—colony growth on hFe solid medium—was found effective to identify altered sensitivity to increased concentration of iron, but not so reliable for the identification of altered phenotype induced by −Fe. While less sensitive to subtle phenotypes, this method selected for mutants with more robust defects, including additional MAPKs like MpkA and MpkB (Jain et al., 2011) and cAMP-PKA signaling, like PkaC1 (Liebmann et al., 2004; Grosse et al., 2008). Importantly, some hFe-sensitive strains exhibited defects also in the presence of other metal high concentration, hinting on a general high metal-related phenotype, and/or upregulation of the vacuolar iron importer *cccA*, suggesting a regulatory defect in iron storage or sensing.

Comparison between the two methods revealed partially overlapping but distinct sets of mutants. Only eight strains were identified by both approaches, while several others were unique to one screen. This divergence likely reflects the distinct cellular processes each assay captures: porphyrin accumulation under −Fe reflects intracellular iron allocation and metabolic bottlenecks, while growth under hFe reveals defects in iron storage, detoxification, or stress tolerance. From a physiological point of view adaptation to iron limitation and iron excess are opposite challenges. Interestingly, the iron sensing transcription factor HapX is essential for both; i.e., screening for both will reveal genes that are exclusively important for one of the challenges or both, e.g., affecting all HapX functions. Thus, both approaches together provide a more comprehensive view of iron homeostasis networks. From a methodological standpoint, each screening has specific advantages. The porphyrin assay is scalable and can reveal early intracellular perturbations, making it suitable for broad initial surveys. However, it requires careful normalization and may yield false positives from general metabolic stress. In contrast, hFe screening is more labor-intensive and sensitive to technical variability (e.g., precipitation, agar composition), but its stringency provides robust phenotypic hits, particularly in signaling mutants affecting iron detoxification or vacuolar function.

A selection of mutants from those identified by one or the other screening, as well as from strains with no obvious defect that were used as control, were subjected to a more thorough characterization that is normally applied to identify iron defects. As some strains exhibited growth defects not only in low or high iron but also in standard condition, a selection of them was also included in the further characterization, to keep into consideration general effects that could be due to reduced growth alone. This characterization included detailed phenotypic analysis on solid medium with various iron concentration also in combination with other stress factors (e.g., hypoxia, light and temperature), siderophore extraction and quantification normalized to biomass, western blot assay targeting HapX protein levels and virulence assay in *G. mellonella*. Unfortunately, it was not possible to establish a clear connection between strains identified with one or the other screening and a consistent outcome in the following characterization. Strains that were identified with altered porphyrin level and growth defect in hFe exhibited more in line results for siderophores production, especially TAFC. Their upregulated HapX expression was more consistent with the strains detected via the porphyrin fluorescence screening, while the broad spectrum of altered phenotypes displayed on plates in various stress conditions was more in agreement with the strains identified by high iron plates screening. HapX levels detected via western blot did not give the impression of being a good evaluation method, as they presented significantly altered value in most of the tested strains, also those used as control. The results from the virulence preliminary assay, however, were found highly encouraging. Two strains identified in both screenings were associated with higher survival in *G. mellonella*, despite the reduced size of this pilot experiment did not allow to reach numbers that can be categorized as significant.

In conclusion, we successfully developed two screening methodologies that are suitable for HTS and can be applied in organisms with no previous knowledge of iron related genes. One strategy, based on porphyrin fluorescence, appeared less stringent but useful to identify a larger number of strains, many of which had impaired levels of proteins already known for their involvement in iron metabolism, like MAPKs or YakA (Jain et al., 2011; van Rhijn et al., 2024). PpIX measurements confirmed further the method reliability. The second strategy, based on colony growth evaluation on high-iron plates, was more selective, identifying strains that presented a relatively strong defect in the presence of high concentration of iron. In this second screening MAPKs were once more highlighted as relevant in iron metabolism, describing for the first time a sensitivity of these kinases not only to low iron concentration, but to high iron as well. Overall, we could speculate that double identification with both screenings might be a little too stringent, excluding strains which could present interesting phenotypes in relation to iron, but it is selecting for genes that presented stronger and consistent outcomes in the characterization assays and have a higher chance to be related to lower virulence potential. The use of one or the other screening methodology, independently, can be used autonomously for targeted search through mutant library.

## Data Availability

The raw data supporting the conclusions of this article will be made available by the authors, without undue reservation.
