# Supplementary material for: Development and optimization of large-scale approaches to identify iron-related genes in Aspergillus fumigatus
Source: Front Microbiol. 2025 Jul 31;16:1646661. doi: 10.3389/fmicb.2025.1646661 (PMC12350462; doi:10.3389/fmicb.2025.1646661)
Supplement: Supplementary file 1 [file Presentation_1.pptx]

## Slide 1
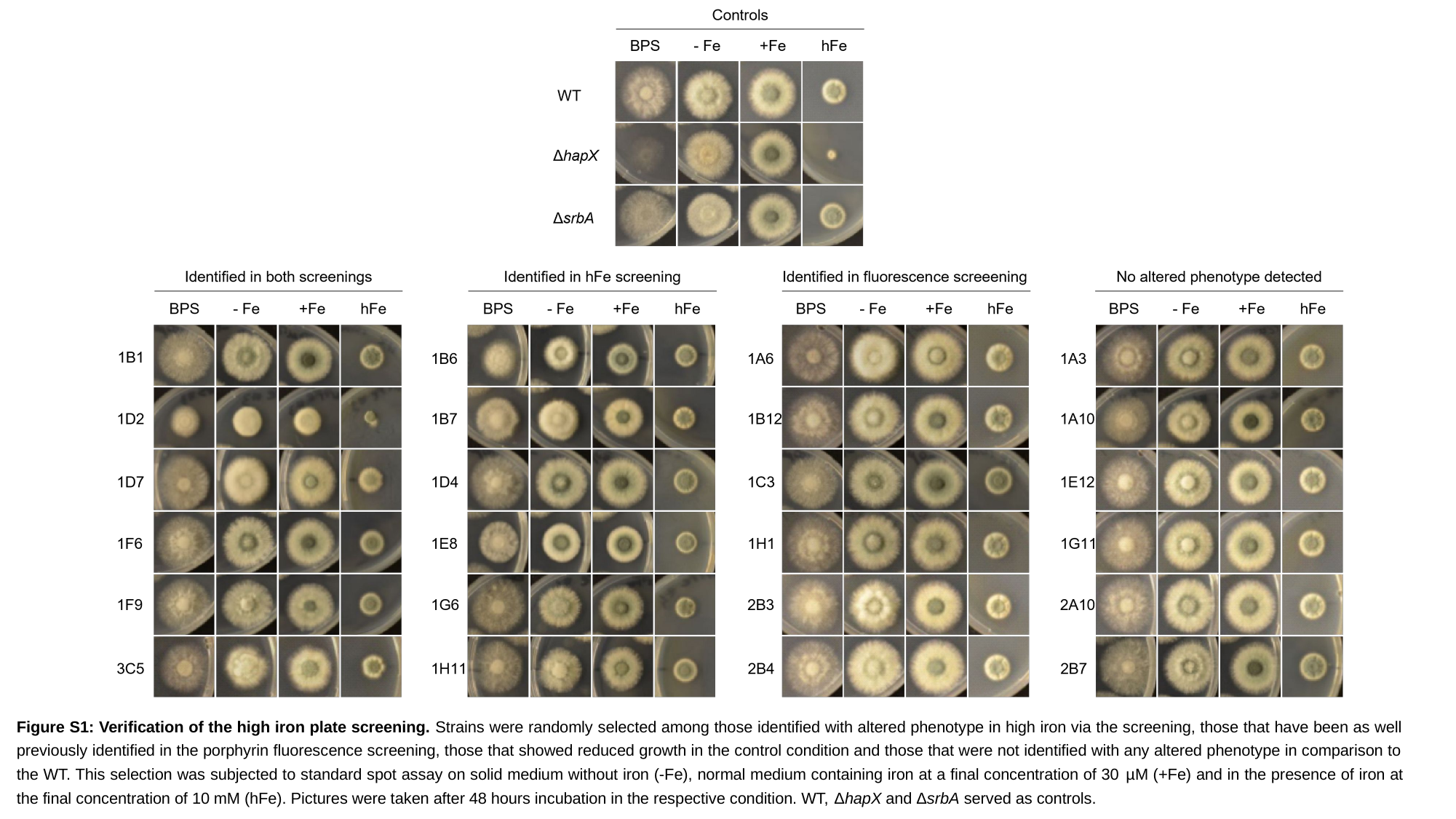

Figure S1: Verification of the high iron plate screening. Strains were randomly selected among those identified with altered phenotype in high iron via the screening, those that have been as well previously identified in the porphyrin fluorescence screening, those that showed reduced growth in the control condition and those that were not identified with any altered phenotype in comparison to the WT. This selection was subjected to standard spot assay on solid medium without iron (-Fe), normal medium containing iron at a final concentration of 30 µM (+Fe) and in the presence of iron at the final concentration of 10 mM (hFe). Pictures were taken after 48 hours incubation in the respective condition. WT, ΔhapX and ΔsrbA served as controls.

## Slide 2
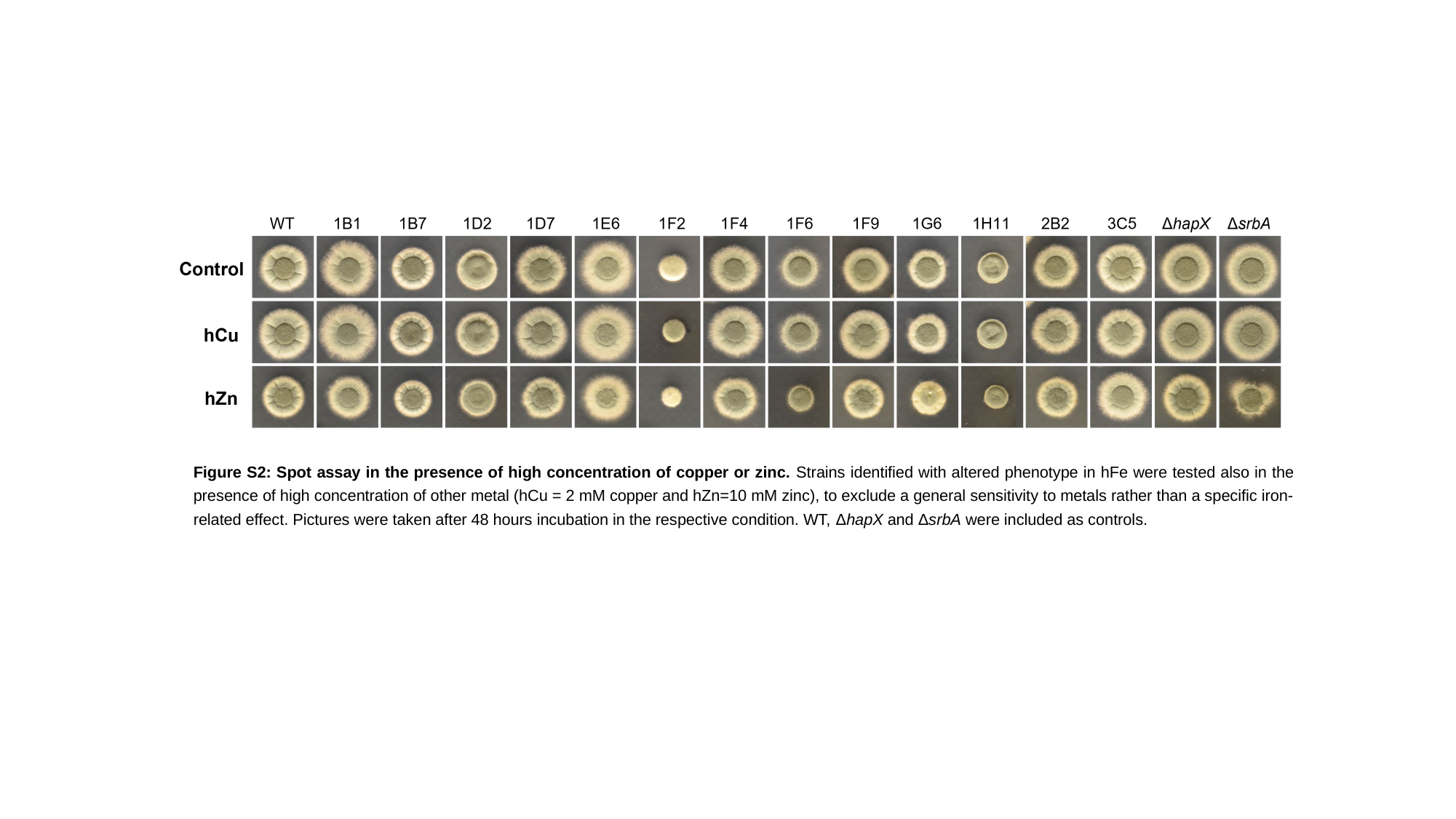

Figure S2: Spot assay in the presence of high concentration of copper or zinc. Strains identified with altered phenotype in hFe were tested also in the presence of high concentration of other metal (hCu = 2 mM copper and hZn=10 mM zinc), to exclude a general sensitivity to metals rather than a specific iron-related effect. Pictures were taken after 48 hours incubation in the respective condition. WT, ΔhapX and ΔsrbA were included as controls.

## Slide 3
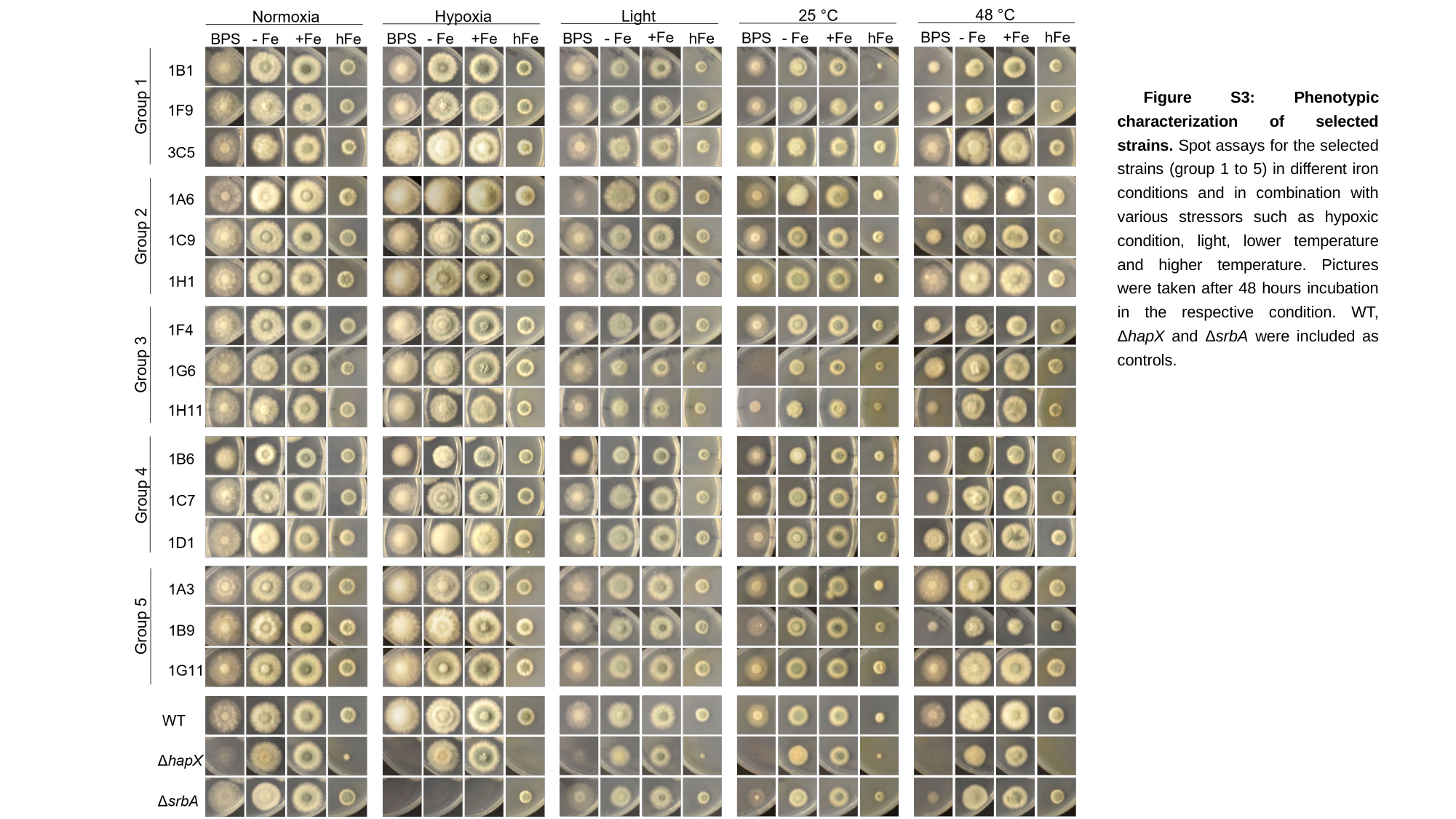

Figure S3: Phenotypic characterization of selected strains. Spot assays for the selected strains (group 1 to 5) in different iron conditions and in combination with various stressors such as hypoxic condition, light, lower temperature and higher temperature. Pictures were taken after 48 hours incubation in the respective condition. WT, ΔhapX and ΔsrbA were included as controls.
